# *Streptococcus suis* Meningitis, Hawaii

**DOI:** 10.3201/eid1512.090825

**Published:** 2009-12

**Authors:** Nahuel Fittipaldi, Tarquin Collis, Bryscen Prothero, Marcelo Gottschalk

**Affiliations:** Université de Montréal, St-Hyacinthe, Quebec, Canada (N. Fittipaldi, M. Gottschalk); Kaiser Moanalua Hospital, Honolulu, Hawaii, USA (T. Collis, B. Prothero)

**Keywords:** Streptococcus suis, human meningitis, bacteria, infection, Hawaii

**To the Editor**: *Streptococcus suis* is a swine pathogen and zoonotic agent responsible for septicemia and meningitis (*1*). *S. suis* is in emergence in some Asian countries. Indeed, this pathogen has been described as the most and second-most common cause of adult meningitis in Vietnam and Thailand, respectively (*2**,**3*). Moreover, during an outbreak in People’s Republic of China in 2005, 39 of 215 patients died from *S. suis* diseases (*4*). On the other hand, only 2 human *S. suis* cases have been reported in the United States (*5**,**6*). Here, we describe a first case of human *S. suis* meningitis in Hawaii.

The patient, a 34-year-old Tongan male with no medical history who worked as a coconut tree trimmer, was singing in his church choir when he developed an acute-onset, global headache and emesis. Upon hospital admission, he described a week of antecedent nonspecific symptoms for which he had taken nonsteroidal antiinflammatory drugs without relief.

On examination, he was afebrile, tired-appearing but alert and with stable vital signs. He presented mild meningismus and photophobia; no rash was observed. Blood tests showed 27,600 leukocytes/µL with 65% neutrophils; 168,000 platelets/µL; hemoglobin 17.3 g/dL; and creatinine 1.4 mg/dL. A computed tomography scan of the head was read as showing substantial motion artifact and a possible cerebral mass. Nuclear **magnetic resonance imaging (MRI)** of the head showed no mass, but T2-weighted images (postgadolinium) suggested both increased grey/white matter contrast consistent with diffuse cortical edema, and vascular congestion/inflammation of the sulci.

Cerebrospinal fluid (CSF) obtained from a lumbar puncture had 2,770 leukocytes/µL with 94% neutrophils; glucose 30 mg/dL; and protein 230 mg/L. A Gram stain of the CSF showed numerous gram-positive cocci, mostly in pairs and short chains (Figure). Empiric intravenous therapy with dexamethasone, vancomycin, and ceftriaxone was administered for possible pneumococcal meningitis.

**Figure:**
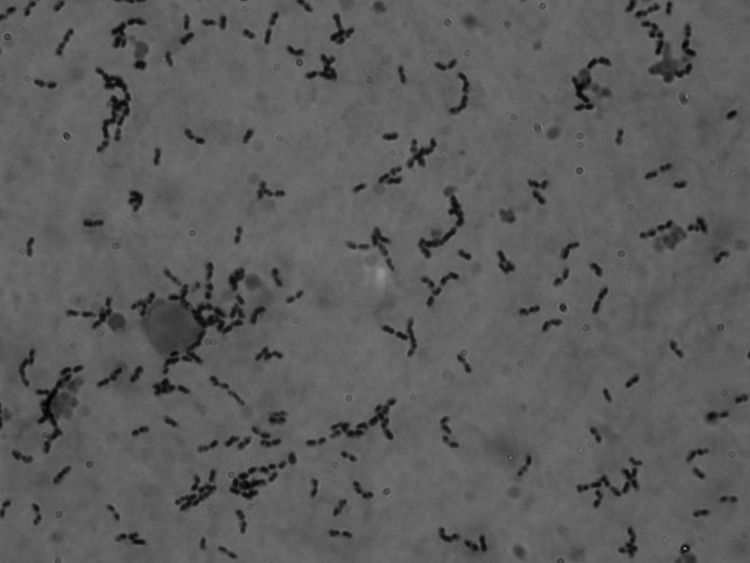
Gram-positive cocci, mostly in pairs and short chains, found in cerebrospinal fluid from a 34-year-old man with *Streptococcus suis* meningitis. The sample was not centrifuged before staining. Original magnification ×1,000.

Blood cultures grew a *Streptococcus* species, later identified by 16S rRNA gene sequencing as being *S. suis*, sensitive to penicillin, vancomycin, and ceftriaxone. The isolate was assigned to serotype 2 by the coagglutination test (*7*) and shown by Western blot to produce suilysin, extracellular protein factor and muramidase-released protein, which are virulence markers often associated with highly virulent strains of Eurasian, but not North American, origin (*1**,**8*). A strain of this phenotype was responsible for a previous US *S. suis* meningitis case, but the patient had been infected in the Philippines (*5*; unpub. data).

Upon identification of the *S. suis* isolate, the patient was questioned about swine contact. He described slaughtering by hand several noncommercially raised pigs over the preceding several weeks for a church-related luau. The patient did not recall any clear incident of mucosal exposure to pig blood or secretions. The exact route of *S. suis* infection for humans is not known. However, most cases have been linked to accidental inoculation through skin injuries (*1*). The patient did not wear gloves, masks, or any other protective equipment during the prolonged process of butchering the pigs, and his exposure to pig blood, skin, and internal organs was extensive. He sustained multiple small cuts on his hands during butchering. No other church members who participated in preparing pigs for the luau became ill.

The patient was treated with ceftriaxone and a 4-day course of dexamathasone. His headache and meningismus improved progressively, and he was discharged after 6 days to complete a 2-week course of intravenous ceftriaxone. However, 1 day after discharge, the patient complained of headaches and mild-to-moderate bilateral hearing loss. He was readmitted; a repeat lumbar puncture showed resolving CSF pleocytosis, and an MRI showed that his prior radiographic findings had normalized. The symptoms, attributed to residual meningeal/cerebral edema, resolved quickly after the reintroduction of steroids. Audiometric testing suggested mild sensorineural hearing loss in the right ear. The patient completed the remainder of his intravenous ceftriaxone course and was discharged on a 2-week course of amoxicillin and oral steroids.

He was again admitted 2 days after completing treatment, with disabling dizziness. On exam he showed new torsional nystagmus, more pronounced with left lateral gaze, consistent with a right peripheral vestibulopathy. An MRI of the head was again normal. Oral dexamethasone promptly resolved his vestibulopathy, and the patient was discharged on a slow steroid taper. After a month, dexamethasone was discontinued. The patient has been asymptomatic since, and his hearing loss has resolved fully.

The role of steroids in treating patients with *S. suis* infection remains unclear, although this case illustrates that the inflammation associated with this infection can be profound and can require prolonged steroid therapy. Since at least 2 cases of relapse have been reported after 2 and 4 weeks of treatment (*1*), prolonged therapy should be considered for infections caused by this pathogen. Hearing loss from *S. suis* meningitis occurs frequently and can be irreversible (*1*). Hawaii’s swine industry is characterized by small herds and a high degree of concentration (*9*). However, the prevalence of *S. suis* among swine in Hawaii is unknown. This case of human *S. suis* meningitis in Hawaii emphasizes the need for these data to be generated and made available. Indeed, this bacterium is increasingly recognized as a significant zoonotic agent in Asia; although it remains a relatively rare cause of human infection elsewhere, persons in close occupational contact with pigs or pork products are at higher risk than others (*1*). Increasing awareness of this disease is expected to help counter human *S. suis* infections.
